# Trends in incidence and treatments of spontaneous subarachnoid hemorrhage- a 10 year hospital based study

**DOI:** 10.1007/s00701-024-06069-z

**Published:** 2024-04-22

**Authors:** Elisabeth Ronne-Engström, Ljubisa Borota, Samuel Lenell, Anders Lewén, Ehab Mahmoud, Christoffer Nyberg, Fartein Velle, Per Enblad

**Affiliations:** 1 Department of Medical Sciences, Section of Neurosurgery, Uppsala University, Uppsala, Sweden; 2 Department of Surgical Sciences, Section of Radiology, Uppsala University, Uppsala, Sweden

**Keywords:** Spontaneous SAH, Aneurysm, Microsurgery, Neurointervention, Stent devices, Stroke incidence, ICP-treatment

## Abstract

**Background:**

Improved endovascular methods make it possible to treat complex ruptured aneurysms, but surgery is still needed in certain cases. We evaluated the effects on the clinical results of the changes in aneurysm treatment.

**Methods:**

The study cohort was 837 patients with spontaneous subarachnoid hemorrhage (SAH) and one or multiple aneurysms, admitted to Dept of Neurosurgery, Uppsala University Hospital from 2012 to 2021. Demography, location and treatment of aneurysms, neurologic condition at admission and discharge, mortality and last tier treatment of high intracranial pressure (ICP) was evaluated. Functional outcome was measured using the Extended Glasgow Outcome Scale (GOSE) Data concerning national incidences of stroke diseases was collected from open Swedish databases.

**Results:**

Endovascular methods were used in 666 cases (79.6%). In 111 (13.3%) with stents. Surgery was performed in 115 cases (13.7%) and 56 patients (6.7%) had no aneurysm treatment. The indications for surgery were a hematoma (51 cases, 44.3%), endovascular treatment not considered safe (47 cases, 40.9%), or had been attempted without success (13 cases, 11.3%). Treatment with stent devices increased, and with surgery decreased over time. There was a trend in decrease in hemicraniectomias over time. Both the patient group admitted awake (*n* = 681) and unconscious (*n* = 156) improved significantly in consciousness between admission and discharge. Favorable outcome (GOSE 5–8) was seen in 69% for patients admitted in Hunt & Hess I-II and 25% for Hunt & Hess III-V. Mortality at one year was 10.9% and 42.7% for those admitted awake and unconscious, respectively.The number of cases decreased during the study period, which was in line with Swedish national data.

**Conclusions:**

The incidence of patients with SAH gradually decreased in our material, in line with national data. The treatment policy in our unit has been shifting to more use of endovascular methods. During the study period the use of hemicraniectomies decreased.

## Introduction

The most important thing when treating a patient with a spontaneous subarachnoid haemorrhage (SAH) is to find and occlude the ruptured aneurysm as quickly as possible. The two main treatment strategies for this- neurointervention and microsurgery- both have advantages and disadvantages, which have been actively debated. Microsurgical clipping of aneurysms has been performed as a standard since neurosurgeons started using intraoperative microscopes. However, it was soon observed that in patients with neurological deficits the brain was more vulnerable to surgical manipulation, and this was shown using the Hunt & Hess classification [13]. It was advised that those with neurological impairment should be treated conservatively, in order to avoid surgical trauma, until they had recovered sufficiently. This improved the surgical outcome, but the obvious problem with this strategy was the risk of rebleeding. Neurointervention, introduced in the 1990s [12], has the advantage of being less invasive, and therefore these patients at higher surgical risk can have their ruptured aneurysms treated early (see e.g. [20]). However, other complications were possible e.g., a rupture of the aneurysm during treatment and could be difficult to control. The International subarachnoid aneurysm trial “ISAT” was a randomized controlled treatment study including ruptured aneurysms in the anterior circulation and the early results showed that outcome after 1 year was significantly better with endovascular treatment [16]. Other studies have supported similar results [14, 15]. Neurointervention was introduced in our centre in 1996 and soon became the preferred treatment of ruptured aneurysms [19, 20] Nevertheless, there will always be a certain number of patients needing emergency surgical treatment of aneurysms and/or hematomas. Over the years, the neurointerventional methods have gradually improved, and the introduction of stents has allowed endovascular treatment of aneurysms with a more complex anatomy. The major drawback with the present stent devices is the need for dual anti-platelet therapy, which could be a disadvantage in the intensive care situation.

In the present study of SAH patients treated from 2012 to 2021 we wanted to analyse changes in demography, treatment policies, neurointensive care treatment and clinical outcome for the patients admitted for ruptured aneurysms. We had three main questions:

- Have there been changes in policies regarding surgical vs. endovascular treatment of ruptured aneurysms?
- Have there been changes in the neurointensive care of patients with ruptured aneurysms regarding the last tier treatment of high intracranial pressure (ICP)?
- What are the clinical results from the acute phase, and the functional outcome after 1 year?

## Methods

All patients with spontaneous subarachnoid haemorrhage admitted to Dept of Neurosurgery, Uppsala University Hospital between Jan 1st, 2012, and Dec 31st 2021 (10 years) were included. The patients came from a geographical area in the middle of Sweden covering a population of 1,98 million 2012 and 2,14 million 2021. Demographic data and clinical information from the acute phase were consecutively entered in a structured administrative database after discharge from neurosurgery. From the database, the patients with single or multiple aneurysms were selected for the present study. Patients who also had an arteriovenous malformation were excluded.

The level of consciousness at admission and at discharge was assessed according to the Reaction Level Scale-85 [24]. The clinical condition at admission was also assessed using Hunt and Hess score [13]. The amount of blood on the first CT-scan was defined by the Fisher scale [11]. Body Mass Index (BMI) was calculated as weight in kilograms divided by the square of height in meters. Mortality during the first year after SAH was recorded.

Computed tomography angiography (CTA) and/or digital subtraction angiography (DSA) was performed to find the bleeding source. Aneurysms were usually treated as soon as possible, except when the patient was in a terminal clinical state already at admission. The final decision on treatment was made in consultation between neurovascular surgeons and neurointerventionists. The policy for aneurysm treatment was to use neurointervention as the first choice when judged to be possible with reasonable risks. Microsurgery was chosen when endovascular treatment was unsuccessful or deemed impossible, when a hematoma required surgical evacuation or when it was decided that dual anti-platelet should be avoided. In this study "aneurysm treatment” refers to treatment of the ruptured aneurysm during the acute phase. The local protocol for neurointervention is described in [6].

During the study period, neurointervention techniques were continuously being developed. In the beginning only coils were used but in 2012 we started using conventional stents and flow diverters. Initially, these were only used occasionally, for example when balloon assisted coiling was not possible and surgery was not considered an option. As stent techniques have improved and our experience increased, the indications for the use of stents have gradually expanded. For example, in the beginning of the study period wide necked aneurysms were operated, but now they are commonly treated with stents. Other devices were introduced in the second half of the study e.g. the woven endobridge device (intrasaccular “web-device”).

The local management protocol for neurointensive care and intermediate care of SAH in the acute phase is described elsewhere [17, 22]. In brief, when consciousness was impaired a ventricular drain was inserted and the patient was mechanically ventilated. Nimodipine was mandatory. DIND (delayed ischemic neurologic deficits) was defined as neurologic deterioration caused by vasospasm when other causes e.g., hydrocephalus and hematomas had been ruled out. DIND was treated by increasing blood volume and blood pressure and also sometimes with intraarterial nimodipine in patients. High intracranial pressure (ICP) was treated with CSF-drainage against a pressure level of 15 mm Hg. Pentobarbiturates and/ or hemicraniectomies were last treatment options when ICP was intractably high. Functional outcome was measured in a structured telephone interview after 1 year, using Extended Glasgow Outcome Scale (GOSE) [26]. Favorable outcome was defined as GOSE 5–8 and poor outcome as GOSE 1–4.

The yearly incidence during the study period of the stroke diseases spontaneous subarachnoid haemorrhage, intracerebral hematoma and cerebral infarction (I60, I61 and I63 according to ICD-10[27]) was taken from Sweden’s National Board of Health and Welfare’s website with open data [1].

### Statistics

**Fig. 1:**
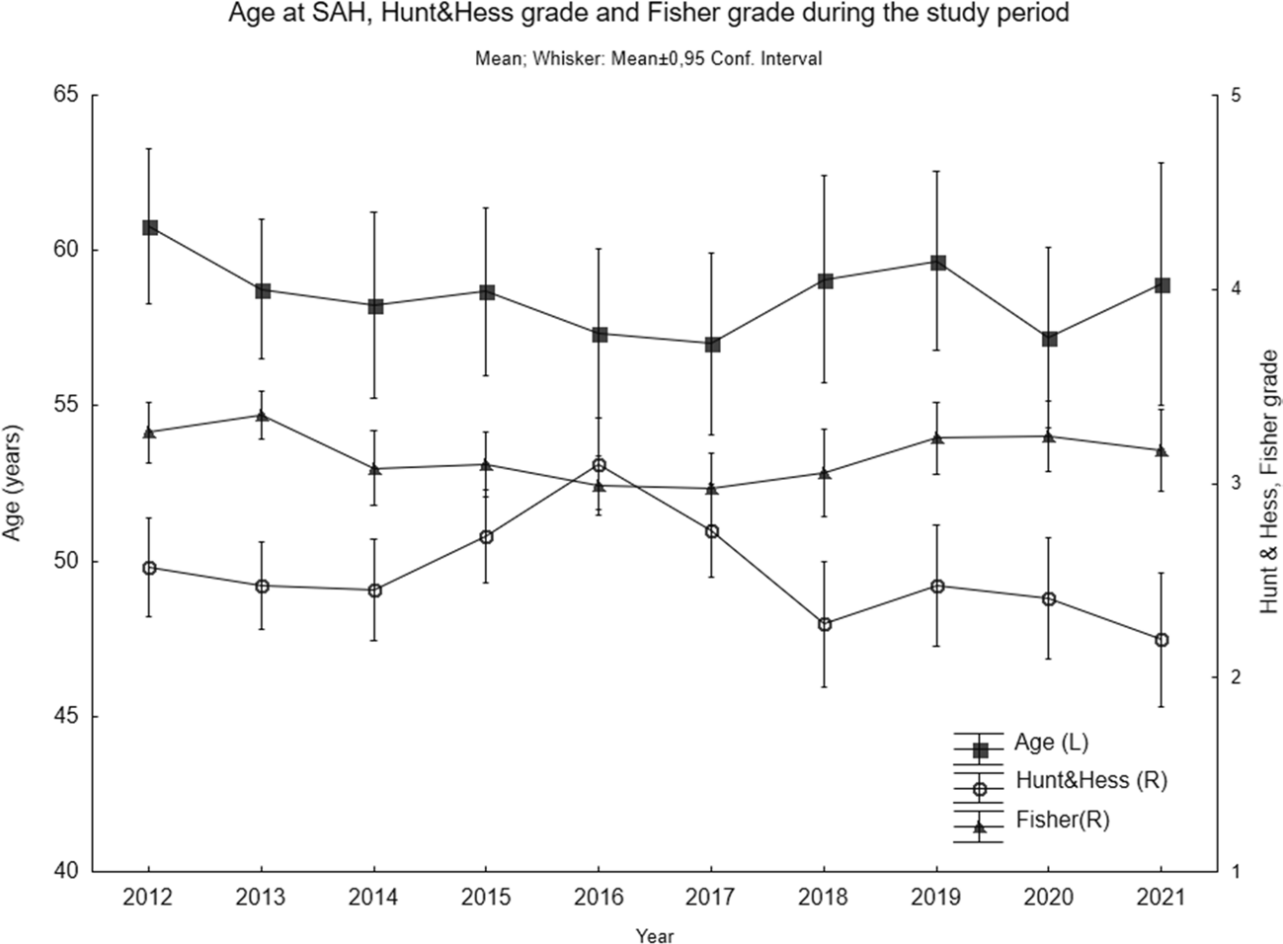
The development during the study period of age at SAH, Fisher grade and Hunt & Hess. The figure shows that there is a shift in the middle years of the period, with lower age, lower Fisher grade and higher Hunt & Hess

The data was not normally distributed and was processed according to this. Median and IQR was used for description. The level of consciousness according to RLS-85 at admission and discharge was compared using Wilcoxon’s rank test for matched pairs. The RLS-85 scale has 8 steps describing level of consciousness. The comparison was done within two groups: those that were conscious at admission (RLS 1–3), and those that were unconscious at admission (RLS 4–8). For the statistical evaluation a 9th step was added representing those that died during the acute phase. In analysing the change in neurointensive care and the functional outcome, 40 patients were excluded that already at admission were in a terminal state due to the severity of the bleeding and that had no aneurysm treatment. χ2- analysis was used to compare distributions. Spearman’s Rank was used for the correlation between age, Fisher grade and Hunt & Hess over time. In Fig.1 mean and 0.95 confidence interval was used to illustrate the changes over time. 

In evaluating functional outcome, univariate analyses was done with Spearman’s Rank and with Mann Whithey-U. A multivariate analyses was done with model building according to Akaike’s information criterion [2]. Variables tested were age, sex, Fisher score, Hunt & Hess at admission and first treatment mode (surgery or neurointervention). To show the distribution of outcome for groups, Hunt & Hess and GOSE were dichotomized into Hunt & Hess I-II vs Hunt & Hess III-V and GOSE 1–4 (poor outcome) vs GOSE 4–8 (favorable). Comparisons of Fisher grade, Hunt & Hess and age between the three treatment groups (surgery and neurointervention with and without stents) were done with Mann Whitney-U. A *p*-value < 0.05 was considered significant. Statistica 13.1, Dell Inc, Tulsa OK, US, was used for analyses.

### Ethical permission

The Swedish ethical review authority granted permission to the study.

## Results

During the 10-year period, 1262 patients with spontaneous SAH were admitted. The distribution of angiographic findings of the source of SAH was: aneurysm (single or multiple) 837 (66.3%), normal angiography 365 (28.9%) aneurysm + AVM 18 (1.4%), AVM/ arteriovenous fistulas (AVF) 18 (1.4%), “other” findings 15 (1.2%) and not performed in 9 (0.7%). The “other” findings included cavernomas, Moyamoya, vasculitis, and dissection. The group with an aneurysm as bleeding source (*n* = 837) was analysed further in this study. The demography of this group, variables at admission and the location of the ruptured aneurysm is shown in Table 1. Since there was a relatively long study period, we plotted the development over time of age at SAH, Hunt &Hess and Fisher grade in Fig. 1. It shows a variation over time with lower age, higher Hunt & Hess and lower Fisher grade in the middle of the study period. There was a significantcorrelation between Fisher grade and Hunt & Hess (0.44) and between Fisher grade and age (0.1) There was no correlation between age and Hunt & Hess.

**Table 1:** This table shows the demography of the aneurysm material

	*N*	%
Women	569	67.9
Age (years)	58.6 ± 13.1	
BMI	26.9 ± 5.7	
RLS-85 at admission	2.5 ± 3.9	
Hunt & Hess	2.6 ± 1.3	
WFNS	2.5 ± 1.4	
Fisher	3.2 ± 0.8	
Ruptured aneurysm		
Proximal ACA and ACom	275	32.8
ICA	202	24.1
MCA	184	22.0
ACA distal of ACom	35	4.2
PCA	19	2.3
BA	76	9.1
VA	47	5.6

*ACA* anterior cerebral artery, *ACom* anterior communicating artery, *ICA* internal carotid artery, *MCA* median cerebral artery, *PCA* posterior cerebral artery, *BA* basilar artery, *VA* vertebral artery

### Aneurysm treatment and SAH incidence

**Fig. 2:**
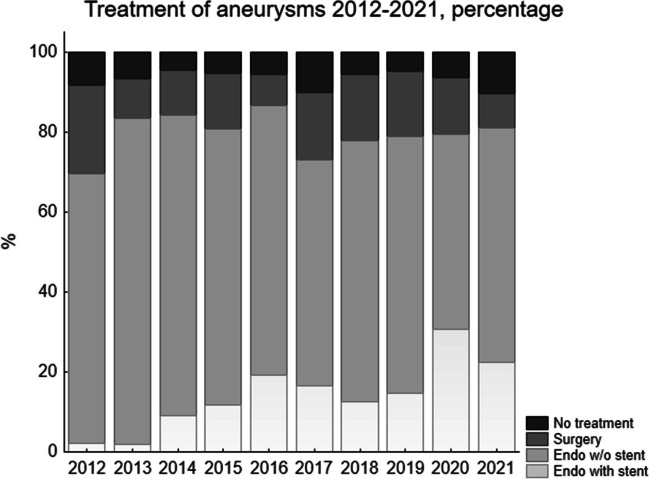
In this figure the distribution of treatments each year is displayed as percentage. There is a trend of decreasing surgical treatment, and at the same time of increased endovascular methods using stent devices

Overall during the whole study period, 79.6% (666) were treated acutely with endovascular methods, with 13.3% of the whole study group (111) involving stents. Surgery was performed in 13.7% (115) and no treatment in 6.7% (56). The indications for surgery were: in 51 cases (44.3%) the need to evacuate a hematoma, in 47 (40.9%) endovascular treatment was considered unsafe, mostly due to a wide aneurysm base involving branches, and in 13 (11.3%), endovascular treatment had been attempted without success. In 3 cases (2.6%) there were other reasons for surgery, e.g., to explore a mycotic aneurysm, remove a giant aneurysm and a suspected rebleeding from a coiled aneurysm. In one case, the indication for surgery was unclear. Of the persons with no treatment, 10 (1.2% of the whole group) had small aneurysms on perforating arteries, that spontaneously occluded. The rest of the patients with no treatment were in a terminal state when admitted. Figure 2 shows the distribution of the treatments. Endovascular methods have a fairly stable percentage, but in an increasing number of these involved stent devices. Table 2 shows basic information for patients treated with neurointervention with and without stents, and with surgery. Patients treated with surgery were significantly younger (*p* < 0.002), in a worse clinical condition at admission (*p* < 0.006) and had more blood on the first CT-scan as defined by the Fisher grade (*p* < 0.0002), compared to those treated with neurointervention without stents. Furthermore, operated patients had significantly more blood on the first CT-scan (*p* < 0.002) compared to patients treated with neurointervention and stent devices, but there was no difference in age or clinical condition at admission. Patients treated with neurointervention and stents were significantly younger than those not treated with stents (*p* < 0.02). In Table 2, the distribution of the three types of treatment for anterior/ posterior circulation is shown and no aneurysm in the posterior circulation was operated during the study decade. Table 2 also shows the rate of hemorrhagic complications and of hemicraniectomies. For surgery, the rate of major bleedings during the procedure is 1.7%. Smaller aneurysm leaks during clipping are not always noted and therefore are not reported here. There were different kinds of intracranial bleedings after treatment. For neurointervention, an increase in already existing minor hematoma was the most common complication, This was also seen after surgery, as well as postoperative hematoma, hemorrhagic venous infarction, cerebellar diachisis and also rebleeding from blood blister aneurysms. The systemic bleedings consisted mostly of hematomas at the femoral artery, but also gastrointestinal bleedings and increased bleedings during other procedures, e.g. insertion of pigtail drain and tracheostomy. Hemicraniectomias were most common in the surgically treated group, which also had the most severe SAH.

**Table 2:** This table shows demographic data, distribution of aneurysms, complications and fuctional outcome after one year for the three treatment groups; surgery and neurointervention with and without stents

Treated aneurysms	Surgery	Neurointervention w/o stent	Neurointervention with stent
*N* =	115	555	111
Age (mean ± SD)	55.0 ± 12.2	59.5 ± 12.9	56.0 ± 14.3
	%	%	%
Women/ Men	63/37	70/30	65/35
Fisher grade I-II / III-IV	13/ 86	21/ 79	14/ 86
Hunt & Hess I-II / III-V	49 / 51	62/ 38	59/ 41
WFNS I-II / III-V	56/44	65/35	60/40
Ruptured aneurysm in anterior/ posterior circulation	100/0	80/20	60/40
Aneurysm leakage during treatment	1.7	4.1	3.6
Intracranial bleeding after treatment	6.0	3.1	9.9
Systemic bleeding	0	2.0	7.2
Delayed Ischemic Neurologic Deficit	24	26	25
Hemicraniectomy	28.8	1.6	3.6
Favorable outcome	54	53	54

**Fig. 3:**
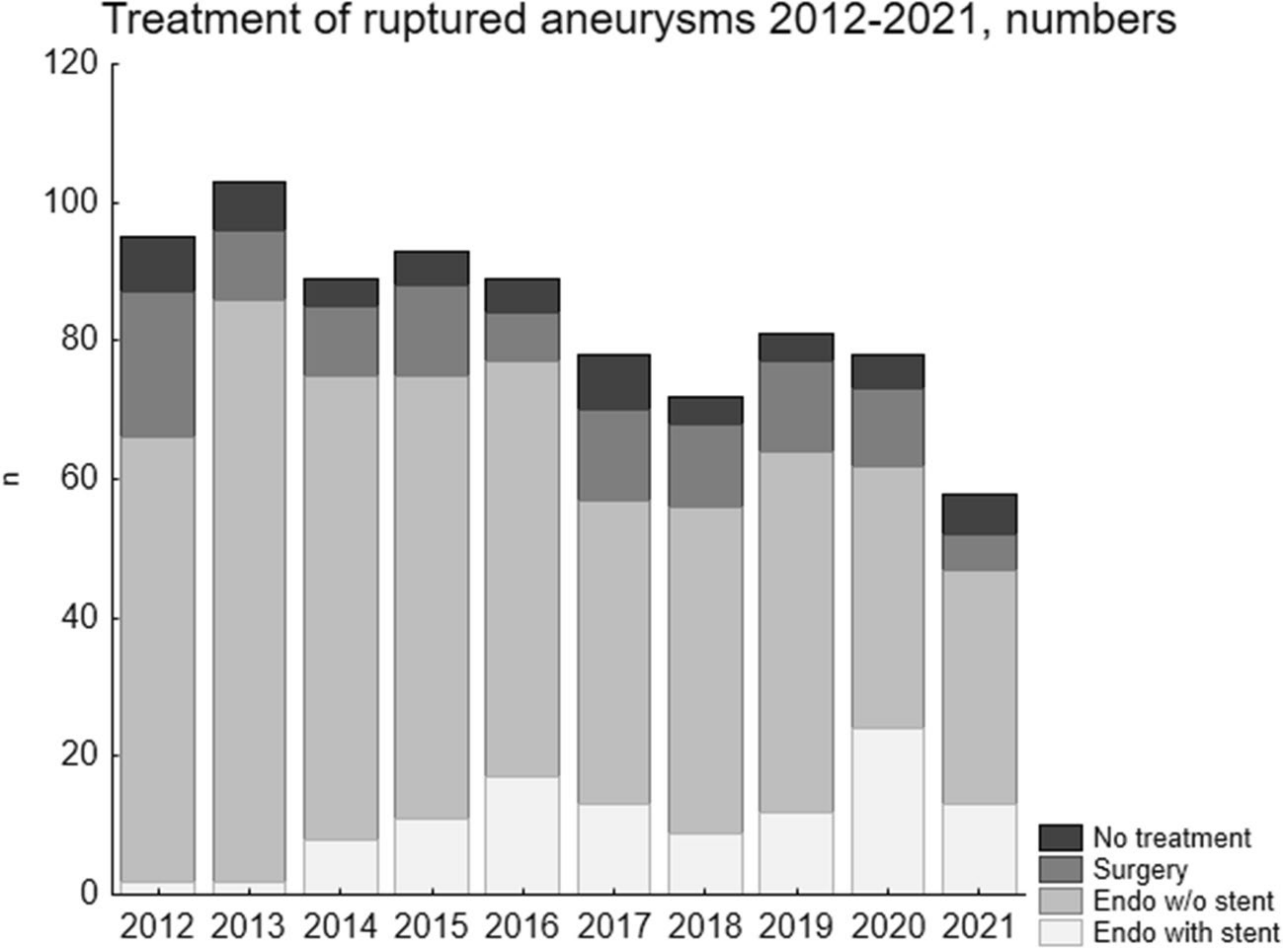
This figure shows the numbers of the different treatments. There is a gradual decrease in the total number of cases during the study period

**Fig. 4:**
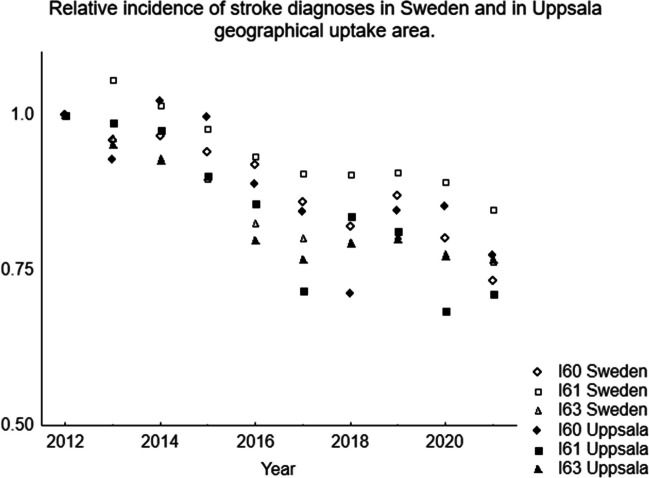
This figure shows the relative incidence of SAH (I60), intracerebral hematoma (I61) and cerebral infarction (I63) on a Swedish national level and on a regional level in Uppsala geographical uptake area. The yearly incidences are related to the incidences at the start of the study period (2012)

There was a steady decrease in new cases admitted to our unit, as shown in Fig. 3. We therefore investigated the development of the incidences for stroke diseases in official national data. In Fig. 4, the relative incidences for SAH, intracerebral haemorrhage and cerebral infarction (I60, I61 and I63 according to ICD-10[27] are plotted with 2012 as the reference level. The incidences are shown for Sweden and for Uppsala geographical uptake area. There is a gradual decrease over the years of all these diagnoses. It also shows that the incidences decrease more in Uppsala geographical uptake area than in Sweden nationally.

### Neurointensive care treatment and clinical course in the acute phase

**Fig. 5:**
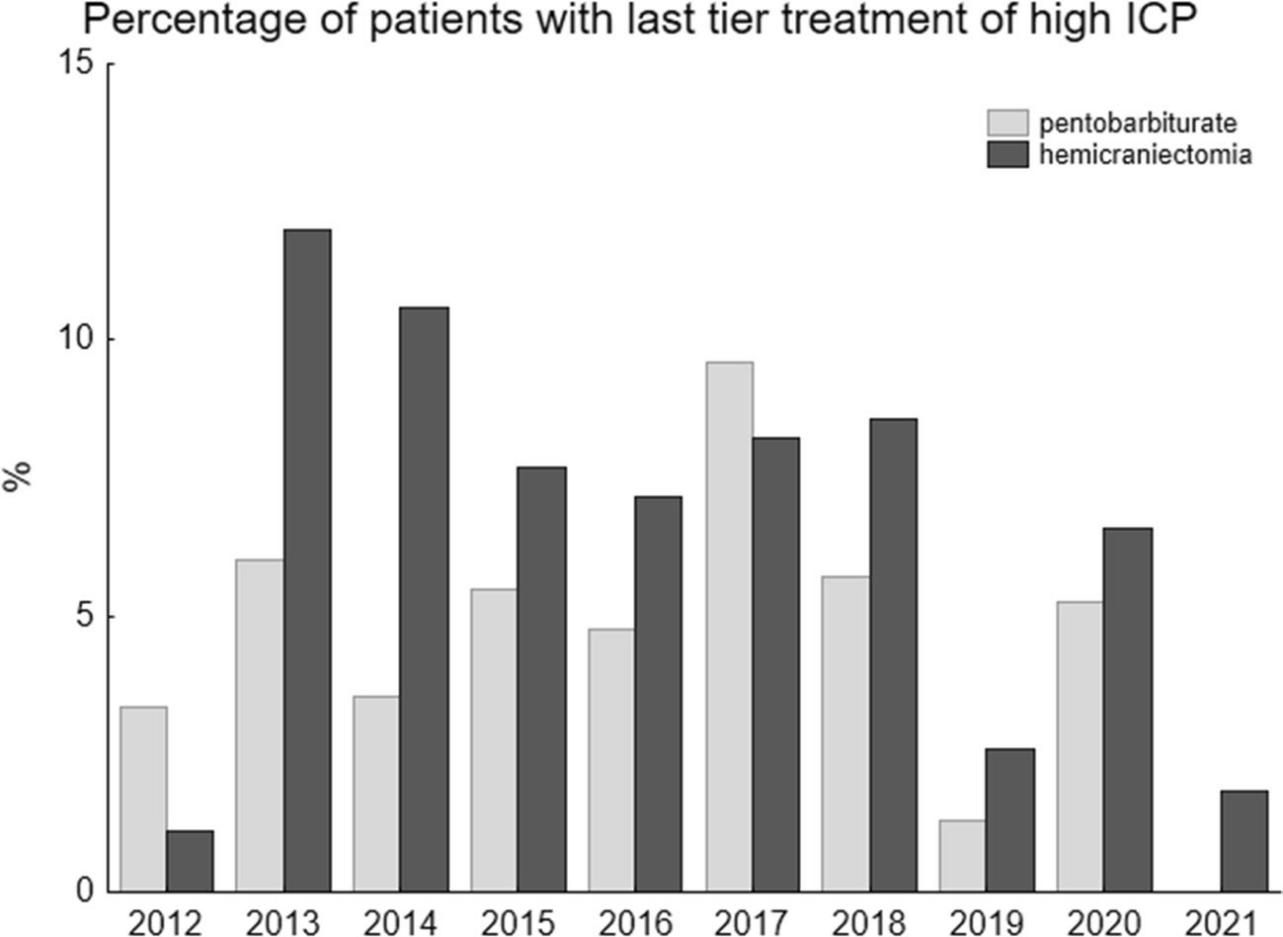
This figure shows the percentage of patients that were treated for aneurysm ruptures and that needed last-tier ICP treatment with either penthobarbiturates or hemicraniectomy (see methods). There is a gradual decrease over the years, except for 2012. Comparison between 2021 and 2013 showed a significant difference for hemicraniectomies

The use of pentobarbiturates and/ or hemicraniectomy was analysed as an indicator of severe problems with high intracranial pressure. The percentage of use per year is shown in Fig. 5. There was an unexpected low percentage of hemicraniectomies 2012. After that we saw a decrease in hemicraniectomies over the years (2020 vs 2013 *p* < 0.05), while there was no significant change in the use of pentobarbiturates. Table 2 shows that the rate of DIND was similar between the three treatment groups but that hemicraniectomy was most common in the surgical group.

The level of consciousness was compared between admission and discharge. They were stratified in two groups: those who were conscious at admission (RLS-85 level 1–3) and those unconscious at admission (RLS-85 level 4–8). Table 2 shows that both groups had a significant improvement of the level of consciousness at discharge. The mortality for after 1 week, 1 month and 1 year is shown in Table 3.

**Table 3:** This table shows the change in level of consciousness according to RLS-85, from admission to discharge. The group was stratified in conscious/ unconscious at admission. The table also shows the mortality at 1 week, 1 month and 1 year

	RLS-85 at admission	RLS-85 at discharge	*P* value
Awake at admission (681)	2 (1; 2)	1 (1; 2)	< 0.0002
Unconscious at admission (156)	5 (4; 7)	3 (2; 9)	< 0.001
Mortality (%)	**1 week**	**1 month**	**1 year**
Awake at admission (681)	3.1	6.9	10.9
Unconscious at admission (156)	26.8	34.3	42.7

### Functional outcome

We had outcome measurements for 91% of the aneurysm cohort. However, the time between the acute phase and the GOSE measurement varied due to radically changed working conditions during the COVID-19-pandemic. Therefore, outcome data collected later than three years after the acute phase was excluded. Also, 40 patients admitted in terminal state (se methods) were excluded. This resulted in functional outcome measurements from 708 patients done at median 383 days (IQR 359;488) after the acute phase. Favorable outcome was seen in 69% for patients admitted in Hunt & Hess I-II and 25% for Hunt & Hess III-V. The univariate analyses showed a significant correlation of GOSE to age (-0.246), Hunt & Hess (-0.387) and to Fisher Score (-0.329). There was no difference in GOSE for treatment mode or for sex. In this analysis treatment mode was defined as surgery or neurointervention (see Methods). The multivariate analysis showed that a model consisting of high Fisher score, high Hunt & Hess and high age best predicted a worse functional outcome (*p* < 0.01 E^−10^). Functional outcome was also calcluated for the three treatment modes (see Table 2) and there was no difference.

## Discussion

### Demography

We found a decreasing number of cases admitted to our department, as well as cases in our geographical region, during the study period. This was in line with the national Swedish situation regarding stroke conditions. The ongoing decrease could indicate a general improvement in vascular health. This could be the result of a higher common awareness and more efficient health care for treating known vascular risk factors, e.g., diabetes and high blood pressure. Better preventive care thanks to National stroke guidelines may also be an important factor. Further, tobacco smoking has decreased in Sweden. According to self reported tobacco habits 11.4% smoked daily 2012 and 6.1% 2021 [18]. In a meta-analysis of the global and regional incidences in SAH between 1980 and 2010, similar results with decreasing SAH incidences were shown [9]. They also correlate the results to a simultaneous decrease in blood pressure and in smoking. In a Finnish study with data from 2003–2014 found a decrease in the incidence of SAH. [23] They suggest that this is the result of working with cardiovascular risk factors on an national level. Another possibility is that the decline in cases shown in our study is the result of shifts in the Swedish population demography. SAH is concentrated in certain age groups, and there have been large variations in nativity in Sweden since the second world war. In Fig. 1, a transient shift in the age at SAH was seen in the middle of the study period, with slightly lower age. At the same time there was also a transient decrease in Fisher grade as well as an increase in the Hunt & Hess score. The age specific incidence of SAH needs to be explored further in future epidemiological studies.

The patients’ demography showed an expected pattern, with a majority of women and a median age of 59 years. The distribution of aneurysms was also what could be expected, with the majority of aneurysms found in the anterior circulation. In the present study we did not monitor earlier diseases, but BMI was noted as a marker for health, since high BMI is thought to increase the risk for ischemic and haemorrhagic stroke [10]. WHO defines overweight as BMI ≥ 25. The median BMI in our study was 26.0. In a recent publication on a national study, BMI was included in models predicting increased mortality after SAH [21] This, and the present results are in line with the literature regarding the importance of BMI for stroke diseases.

### Aneurysm treatment

In the first half of the study period, the aneurysms were always treated with coils only, when not clipped. Some of these patients came back later (data not included) for additional treatment, which was done with coils or with stents. This is in line with the experiences from the ISAT study in which 17.4% of endovascular treated patients needed an additional treatment while 3.8% of surgically treated patients needed additional treatment of the aneurysm [7]. In the later part of our study period, stents were more often used in the acute phase. We observed that no aneurysm in the posterior circulation was operated on in the present study, and that use of acute stents was more common in that location. This could indicate that aneurysms in the posterior circulation had a more complete treatment in the study period compared to earlier. However, this was not analyzed further. This is not only a result of improvement of stents, but also of accumulated experience within the team. The neurovascular team consists of 4 vascular neurosurgeons and 4 neurointerventionists of which 2 also are neurorsurgeons. Treatment decisions were in practically all cases taken after discussions between neurosurgeons and neurointerventionists about what would be the best for the patient. This way of working also made it easier to increase the groups experiences regarding stent use. The most important drawback of the use of stents in the acute phase is mandatory dual anti-platelet therapy. As seen in Table 2, patients with stents had a higher rate of postprocedure intracranial as well as systemic hemorrhage. Still, the percentage of patients in favorable outcome was similar between the three treatment groups. Recently new intrasaccular devices for endovascular treatment of aneurysms with wide necks were developed that do no require dual anti-platelet treatment [8]. There is also an ongoing development of the coiling-techniques and it is possible that in the future the need for stents will decrease. However,with an assumed continuous improvement of the endovascular techniques, probably even fewer aneurysms need surgical occlusion. It seems though, that even if all aneurysms could be treated endovascularly in the future, there would still be a need for surgical evacuation of hematomas, and probably clipping of aneurysms at the same time. Training new vascular neurosurgeons is already a problem due to the decrease in surgical procedures, which in turn is associated with the decreasing incidence of SAH and improved neurointerventional methods. Nevertheless, from the patients’ perspective, it can never be ethical to perform a surgery just for the sake of training when less invasive treatments are available. Possibly new strategies can be developed using hybrid operation theatres equipped for both neurointervention and neurosurgery. For example, a ruptured aneurysm could be secured with neurointervention while the patient is prepared for evacuation of a hematoma.

### Clinical course in the acute phase and neurointensive care treatment

In order to see the direct effect of our management strategy on the study patient group we measured the neurological condition at admission and discharge in both patients who were conscious and unconscious on admission. We found that overall on discharge the level of consciousness had improved for both groups. We suggest that the extent of the acute brain injury relates better to the change in consciousness during the acute phase than to the long-term functional outcome, since this in turn depends on many factors besides the acute brain injury.

In an earlier study from our department our experiences of hemicraniectomies as a last-tier treatment of high ICP in patients with aneurysmal SAH was described [5]. The results showed that 10% had intractable high ICP in spite of standardized treatment protocols, hemicraniectomia effectively reduced ICP, saved life and improved outcome. During the present study period the use of hemicraniectomies decreased. We saw a temporal relation to increased use of stents but did not have data to study the possible causative relationship. It could be speculated that more frequent surgical treatments in the earlier years resulted in more complications. It is generally known that the brain after SAH is vulnerable to additional injury from surgical manipulation, and therefore early surgery should avoided when the patient is in a bad clinical condition. In a recent analysis of a Swedish nationwide prospective study, 25% of those treated with surgery had adverse events associated with the operation, and this was related to a worse outcome [3]. In the same study, 13% of those treated with endovascular techniques had complications in association with the treatment [4]. This suggests that surgical treatment could be more traumatic, although there is an obvious selection bias. There is another possible contributing factor for the decreasing need for hemicraniectomies as a last-tier ICP treatment associated with increased neurointerventional treatments. These treatments often require single or dual anti-platelet therapy, which could help reduce vasospasm and delayed ischemic neurological deficits. Zhao et al. [28] in a review with a meta-analysis concluded that anti-platelet therapy after endovascular treatment was associated with reduced mortality, better functional outcome, and a decrease in the incidence of DCI. In an early randomized controlled study on acetylsalicylic acid and SAH no beneficial effect was seen [25]. In that study, 2/3 of the patients were treated with surgery and it is possible that the ASA could not compensate for the surgical trauma. Considering the rapid change in treatment policies, more studies on anti-platelet therapy are needed.

### Functional outcome

The outcome was in line with our earlier results [19, 20]. However, due to the variation in when the outcome measurement was done in those papers, a strict comparison cannot be performed. Multivariate analysis showed that a model consisting of more blood on the first CT-scan, worse clinical condition and high age best predicted a worse outcome. Treatment mode was not included in the best predicting model, and did not result in a significant difference in the functional outcome in the univariate analysis. However, the study was not designed to compare treatment modes but to evaluate the results from our present treatment policy which includes the choice of treatment.

### Strengths and limitations

The strength with our study is that it is based on our administrative database, which means that all patients that were referred to us during the study period with the diagnosis of interest were included.

Since the preferred treatment was endovascular, and surgery only was done in selected cases the data did not allow for comparison of the methods, However, this was not the intention of the study. Another weakness is that we did not study more in detail complications due to the different treatments. We focused more on the actual results.

We found changes in different variables e.g. type of aneurysm treatment, last-tier ICP treatment and incidence of SAH. However, we could just observe the temporal relation but we do not have data for studying the causative relations. Other factors are probably important too e.g. changes in treatment policies in neurointensive care.

## Conclusions

We found that the incidence of patients with SAH was gradually decreasing, in line with national data. The treatment policy has been shifting to more use of endovascular methods. During the same period the need for hemicraniectomies as a last-tier ICP treatment decreased, possibly due to the use of less invasive aneurysm treatments. The functional outcome was best predicted by a model consisting of age, clinical condition at admission and amount blood on the first CT-scan.

## Data Availability

Not available according to the ethical approval.

## Code availability

NA.
